# PTV margin definition in hypofractionated IGRT of localized prostate cancer using cone beam CT and orthogonal image pairs with fiducial markers

**DOI:** 10.1186/s13014-014-0229-z

**Published:** 2014-11-11

**Authors:** Christoph Oehler, Stephanie Lang, Peter Dimmerling, Christian Bolesch, Stephan Kloeck, Alessandra Tini, Christoph Glanzmann, Yousef Najafi, Gabriela Studer, Daniel R Zwahlen

**Affiliations:** Department of Radiation Oncology, University Hospital Zurich, Zurich, Switzerland; Department of Radiation Oncology, Hospital Graubuenden, Chur, Switzerland

**Keywords:** Prostate cancer, Radiation therapy, IGRT, VMAT, Cone beam CT, PTV margin definition, Hypofractionation

## Abstract

**Purpose:**

To evaluate PTV margins for hypofractionated IGRT of prostate comparing kV/kV imaging or CBCT.

**Patients and methods:**

Between 2009 and 2012, 20 patients with low- (LR), intermediate- (IR) and high-risk (HR) prostate cancer were treated with VMAT in supine position with fiducial markers (FM), endorectal balloon (ERB) and full bladder. CBCT’s and kV/kV imaging were performed before and additional CBCT’s after treatment assessing intra-fraction motion. CTV_P_ for 5 patients with LR and CTV_PSV_ for 5 patients with IR/HR prostate cancer were contoured independently by 3 radiation oncologists using MRI. The van Hark formula (PTV margin =2.5*Σ* +0.7*σ*) was applied to calculate PTV margins of prostate/seminal vesicles (P/PSV) using CBCT or FM.

**Results:**

172 and 52 CBCTs before and after RT and 507 kV/kV images before RT were analysed. Differences between FM in CBCT or in planar kV image pairs were below 1 mm. Accounting for both random and systematic uncertainties anisotropic PTV margins were 5-8 mm for P (LR) and 6-11 mm for PSV (IR/HR). Random uncertainties like intra-fraction and inter-fraction (setup) uncertainties were of similar magnitude (0.9-1.4 mm). Largest uncertainty was introduced by CTV delineation (LR: 1-2 mm, IR/HR: 1.6-3.5 mm). Patient positioning using bone matching or ERB-matching resulted in larger PTV margins.

**Conclusions:**

For IGRT CBCT or kV/kV-image pairs with FM are interchangeable in respect of accuracy. Especially for hypofractionated RT, PTV margins can be kept in the range of 5 mm or below if stringent daily IGRT, ideally including prostate tracking, is applied. MR-based CTV delineation optimization is recommended.

## Background

Image-guided radiation therapy (IGRT) is the preferred method for curative treatment of localized prostate cancer and is associated with improved outcome and reduced toxicity [1]. IGRT using cone-beam computed tomography (CBCT) or kilovoltage (kV/kV) gold fiducial marker (FM) imaging in combination with conformal radiation therapy (RT) techniques such as intensity-modulated radiotherapy (IMRT) or volumetric modulated arc therapy (VMAT) potentially allow smaller treatment margins and to escalate dose to the prostate [2].

PTV margins have been reduced from 10 mm in the pre-IGRT-era to 3-5 mm in the IGRT-era [3,4]. There is little consensus on the magnitude of PTV margin for RT of the prostate mainly due to inter-institutional technical issues and the preferred IGRT modality [5].

To compute the magnitude of a PTV-margin that allows the CTV receive the prescribed dose with a clinically acceptable and specified probability, statistics of all uncertainties in the treatment process chain should be known [6,7]. Geometrical uncertainties in RT include both treatment preparation variations and execution uncertainties. They both can be systematic such as equipment maladjustments, planning setup uncertainties, and target volume delineation or random such as treatment setup uncertainties, inter- and intra-fraction organ motion [7].

The aim of this work was to investigate PTV margin determination in the IGRT-era with endorectal balloon (ERB) calculating systematic and random treatment uncertainties. Furthermore, the IGRT modalities CBCT and kV/kV FM imaging were compared with each other in the context of PTV margin determination.

## Patients and methods

### Study design and patient selection

From January 2009 to April 2012, 20 non-consecutive patients with histologically proven low- (LR), intermediate- (IR) or high-risk (HR) adenocarcinoma of the prostate were treated according to the CHHIP (Conventional or Hypofractionated High Dose Intensity Modulated Radiotherapy for Prostate Cancer) protocol [8], and according to randomization VMAT (RapidArc®) was used to a dose of 74Gy/37f (n = 14), 60Gy/20f (n = 1) or 57Gy/19f (n = 5) at the University Hospital Zürich, Switzerland [9]. Hypofractionation schedules were used only for patients randomized within the CHHIP trial (open between 2010 and 2011 at Zurich) and not off-study, since hypofractionation was not standard at that time. Planning CT scans, kV/kV images and CBCT’s of the 20 patients were retrospectively analysed with ethics approval (Department of Radiation Oncology, University Hospital Zurich).

### Target definition and treatment planning

Two weeks prior to CT simulations, 3 FM were placed into the prostate under rectal ultrasound guidance. Non-contrasted planning CT simulation with axial slices thicknesses of 2.0 mm was performed in supine position by use of a leg holder immobilization device, empty rectum with endorectal balloon (ERB) and full bladder. Reference points were tattooed on the skin.

CTV was defined as prostate (P) only for LR, prostate plus 1/3 of the seminal vesicles (PSV) for IR or the prostate plus the seminal vesicles for HR cancer patients. In order to evaluate the delineation error of prostate only or prostate/seminal vesicles, the CTV_P_ for 5 pts with LR and CTV_PSV_ for 5 pts with HR prostate cancer were contoured independently by 3 experienced radiation oncologists using non fused MR images with rectal coil. No formal contouring training was performed.

### Treatment

Daily localization of the prostate position was performed using FM before treatment. CBCT was performed on day 1 to 3, then once every week (hypofractionation: 6 × 6 CBCT, normofractionation: 14 × 9-10 CBCT). Additional in-between CBCT were not used for evaluation. For 10 patients (57Gy/19f (n = 1), 74Gy/37f (n = 9)) an additional CBCT was performed after each fraction to assess intra-fractional motion once a week (1 × 4 CBCT, 9 × 7 CBCT). Due to patient’s distress, failure of CBCT or correlation function, not all post-treatment CBCT could be used for evaluation. On day 1 the isocenter after CBCT based corrections was marked on the patient skin. Online isocenter positioning was performed based on FM using CBCT – as indicated above - or kV image pairs. kV/kV images were performed before each fraction except when a weekly or in-between CBCT was applied. On days with post-treatment CBCT a kV/kV image pair was performed for comparison reason (14 × 37 kV/kV +5 × 19 kV/kV +1 × 20 kV/kV =633 kV/kV – 172 kV/kV (pre-CBCT days) +67 kV/kV (post-CBCT days) =528 kV/kV – 21 kV/kV (in-between CBCT) =507 kV/kV).

### Statistical analysis

The van Herk formula M =2.5*Σ* +0.7*σ* was used for PTV margin calculation, where Σ represents the systematic and σ the random uncertainty. Random uncertainties are statistical fluctuations whereas systematic uncertainties are often due to a problem which persists throughout the entire treatment. We calculated systematic uncertainty for intra-fractional motion, contouring as well as setup. Random uncertainties were calculated for intra-fractional motion and patient setup. Total *Σ* was then calculated: *Σ* = (*Σ*^2^_contour_ + *Σ*^2^_patient setup_ + *Σ*^2^_intrafraction motion_)^1/2^ as well as total *σ*: *σ* = (*σ*^2^_patient setup_ + *σ*^2^_intrafraction motion_)^1/2^. Statistical analysis was performed with MATLAB, version 11 (Mathworks, MA, U.S.A.). We used a *σ*_p_ – where *σ*_p_ is the standard deviation of the normal dose penumbra - of 3 mm assuming the worst case (cranial-caudal direction) because for VMAT *σ*_p_ is larger in the left/right as well as anterior/posterior direction compared to the cranial/caudal direction. Furthermore, changing the *σ*_p_ influences only the random error and results in a small change of the margin.

The online match was based on FM. Additionally offline matches based on bony anatomy and ERB (kV/kV image pairs and CBCT), as well as soft prostate tissue (CBCT) were performed to compare different matching techniques. The setup uncertainty was calculated for each of the different matching techniques in respect to the online performed FM match. To assess the difference between FM match in kV image pairs and CBCT the two imaging modalities were performed strictly consecutively and shifts were compared. To assess intra-fraction prostate motion, differences between FM position in pre-treatment and post-treatment CBCTs were established.

**Figure 1:**
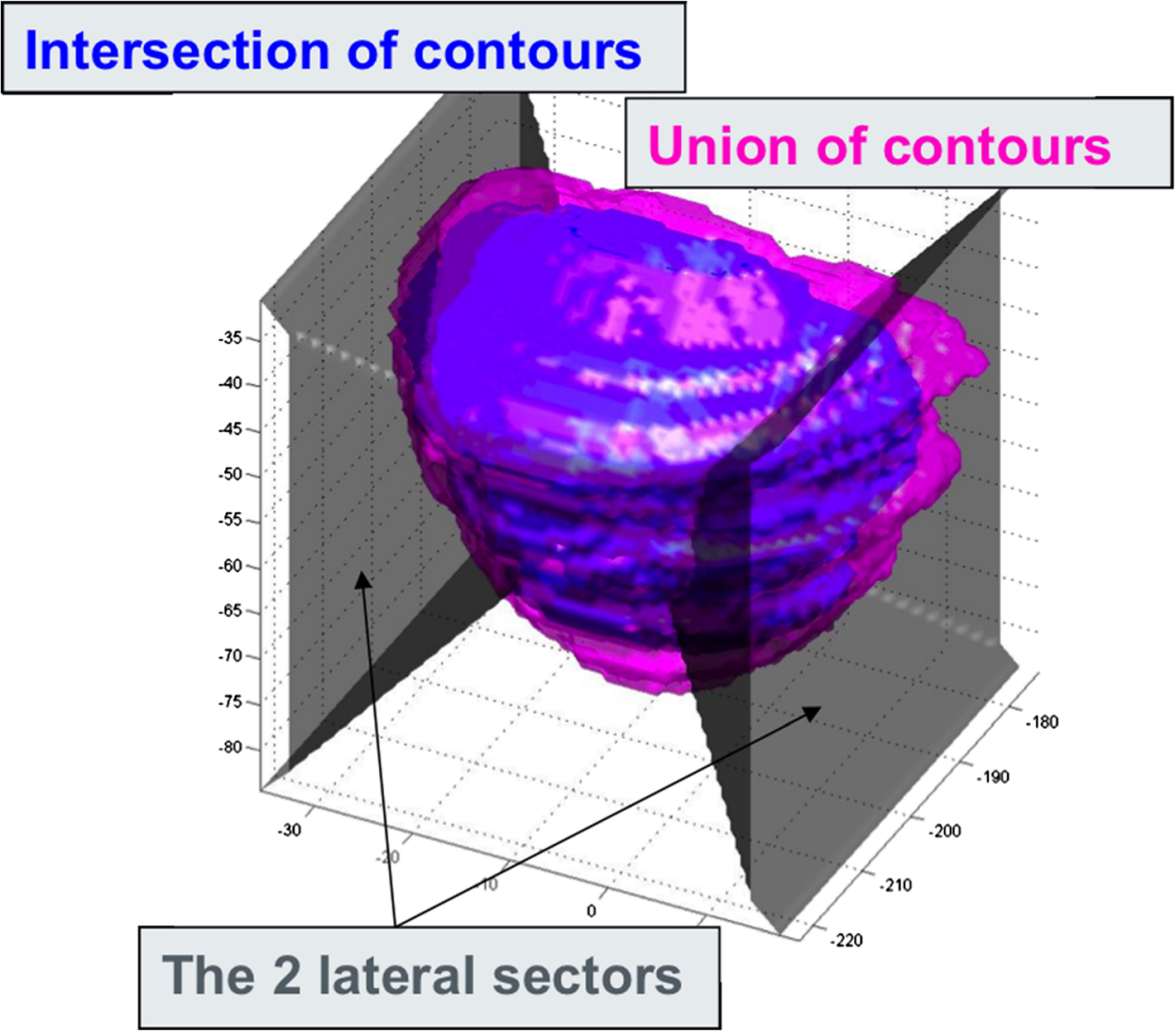
**Depiction of the mean differences in vertical, lateral and longitudinal directions between the intersections (blue) and the unions (pink) of the three CTVp as contoured by three radiation oncologists.** The prostate volume was divided into 6 subsections (anterior, posterior, left, right, cranial, caudal).

The contouring uncertainties of the CTV_P_ and the CTV_PSV_ were determined by comparing intersection and union of 2 corresponding contours using a self-developed analysis script in Matlab (Mathworks): The prostate volume was divided into 6 subsections (anterior, posterior, left, right, cranial and caudal) (Figure 1). For each subsection the mean distance between the union and the intersection was calculated. The mean and standard deviation (SD) between the pairwise comparisons were calculated. Wilcoxon-Mann-Whitney-Test was used to compare contouring uncertainties for P and PSV.

## Results

### Setup (inter-fractional) uncertainty

**Figure 2:**
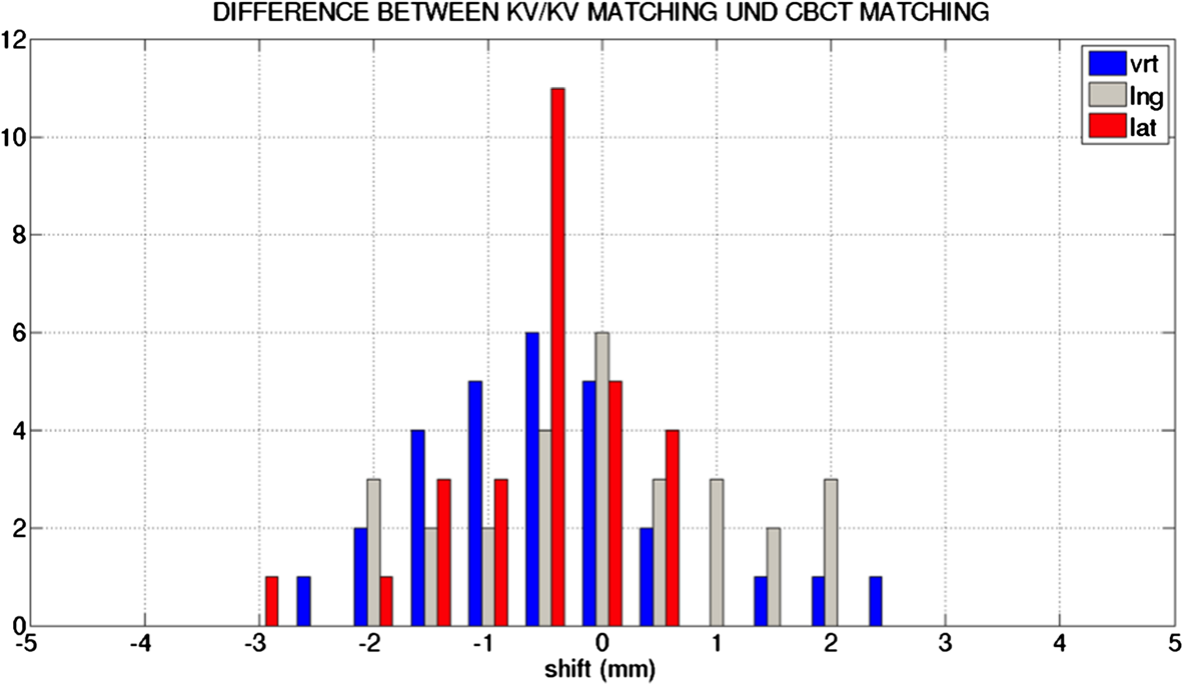
**Bar diagram of differences in vertical (vrt), longitudinal (lng) and lateral (lat) direction between CBCT and kV/kV setup matching on fiducial markers (N =10).**

172 and 52 CBCT before and after RT and 507 kV/kV images before RT were analyzed. The setup uncertainty was calculated for the matching on different surrogates in kV images pairs as well as CBCT using CBCT FM match as reference. The mean systematic and random setup uncertainties for each modality are shown in Tables 1 and 2. The difference in setup using FM between CBCT and kV/kV was usually below 2 mm (Figure 2). Matching on FM in kV/kV images or matching on soft tissue in CBCT lead to systematic and random uncertainties below 1 mm. Bony anatomy match as well as balloon match increased the systematic as well as the random uncertainty. This was more pronounced if kV/kV image pairs were used compared to the use of CBCT. Large systematic as well as random uncertainties in longitudinal direction were found with the use of the ERB as a surrogate for prostate position since reproducible placing of the ERB was not possible.

**Table 1:** **Systematic Σ and random σ setup error depending on surrogate used for matching kV/kV images to the reference CT**

		***Σ*** **(mm)**	***σ*** **(mm)**
Bones	vrt	1.78	1.97
	lng	1.39	2.30
	lat	0.77	0.85
Balloon	vrt	1.53	1.36
	lng	3.20	3.15
	lat	1.70	1.35
FM	vrt	0.88	0.71
	lng	1.16	0.46
	lat	0.46	0.37

**Table 2:** **Systematic Σ and random σ setup error depending on surrogate used for matching the CBCT to the reference CT**

		***Σ*** **(mm)**	***σ*** **(mm)**
Bones	vrt	1.92	1.87
	lng	1.72	1.69
	lat	0.61	0.90
Balloon	vrt	1.68	1.82
	lng	1.20	1.66
Soft tissue	vrt	0.77	1.09
	lng	0.36	0.17
		0.49	0.93

Fiducial marker matching in CBCT was taken as a reference.

### Intra-fractional motion uncertainty

**Figure 3:**
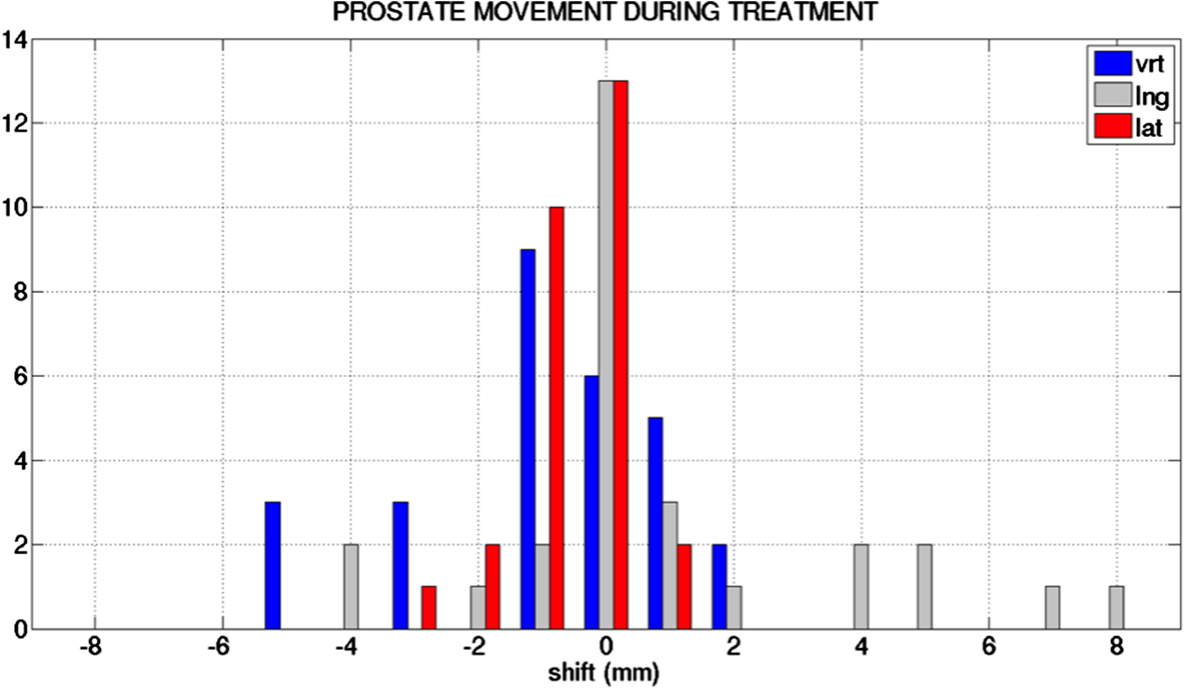
**Bar diagram of intra-fraction motion in vertical (vrt), longitudinal (lng) and lateral (lat) direction during treatment (N =10).** Intra-fraction motion was determined by fiducial marker difference between pre- and post- CBCT.

For intra-fractional motion uncertainty calculation of the prostate FM shift of pre- and post-treatment CBCT’s were compared with each other (time between 3 to 6 min). Maximum FM shifts reached 5 mm in vertical and 8 mm in longitudinal direction (Figure 3). The systematic intra-fractional uncertainty was 1.39 mm (vrt), 1.36 mm (lng), 0.92 mm (lat) and the day to day random uncertainty was 1.62 mm (vrt), 1.39 mm (lng), 0.97 mm (lat), respectively.

### Contouring error

Systematic contouring uncertainties were larger for PSV than for P (*p* =0.012). The mean systematic uncertainty was largest in the longitudinal axis as shown in Table 3. In vertical direction the uncertainty was found to be larger in anterior (1.98 mm (P), 3.49 mm (PSV)) than posterior (1.04 mm (P), 1.58 mm (PSV) direction.

**Table 3:** **Average contouring errors for prostate (P) or prostate with seminal vesicles (PSV) in anterior (vrt/anterior), posterior (vrt/posterior), longitudinal (long) and lateral (lat) direction**

	**P (mm)**	**PSV (mm)**
vrt	1.52	2.57
vrt/anterior	1.98	3.49
vrt/posterior	1.04	1.58
Long	2.03	3.20
Lat	1.68	2.52

### PTV margin (according to van Herk formula)

The calculated PTV margins can be found in Tables 4 and 5. The margin for P only was calculated to be 7.2 mm in anterior, 5.5 mm posterior, 7.1 mm longitudinal, and 5.5 mm in lateral direction. PTV margins were larger for PSV volumes than for P only volumes due to the contouring differences. The difference was largest in longitudinal direction (2.5 mm). The contouring uncertainty contributed most to the PTV margin compared with the intra-fractional and setup uncertainties. Matching on soft tissue in the CBCT or on FM in kV/kV image pairs increased the margin by less than 0.7 mm. Matching on the ERB required an additional margin of up to 5.4 mm.

**Table 4:** **Margins needed to be applied for patients with low risk prostate cancer when matching on fiducial markers (FM), soft tissue (ST), bones (B) or balloon (ERB)**

	**M (mm) CBCT**	**ST (mm) CBCT**	**B (mm) CBCT**	**Ba (mm) CBCT**	**M (mm) kV**	**B (mm) kV**	**Ba (mm) kV**
Anterior	7.18	7.69	9.45	9.07	7.67	8.64	8.64
Posterior	5.47	6.09	8.20	7.75	6.10	7.27	7.27
Long	7.09	7.16	9.00	8.32	7.79	12.48	12.48
Lat	5.47	5.88	5.95	6.10	5.65	7.58	7.58

**Table 5:** **Margins needed to be applied for patients with high risk prostate cancer when matching on fiducial markers (FM), soft tissue (ST), bones (B) or balloon (ERB)**

	**M (mm) CBCT**	**ST (mm) CBCT**	**B (mm) CBCT**	**Ba (mm) CBCT**	**M (mm) kV**	**B (mm) kV**	**Ba (mm) kV**
Anterior	10.52	10.94	12.28	11.99	10.88	12.18	11.62
Posterior	6.39	6.94	8.85	8.44	6.94	8.68	7.99
Long	9.67	9.72	11.23	10.71	10.19	11.24	14.23
Lat	7.39	7.76	7.80	7.95	7.53	7.88	9.11

For low risk prostate cancer patients the prostate only was treated. For high risk prostate cancer patients the prostate and the seminal vesicles were treated.

## Discussion

In the IMRT-IGRT-era, PTV margin determination has become of crucial importance [3,10]. We found a PTV margin between 5-8 mm for the P (LR) and between 6-11 mm for the PSV (IR/HR). A similar prostate PTV margin of 7 mm was determined by van Herk and colleagues though intra-fractional motion was not considered in their calculation and no ERB was used [11]. PTV margins used at the institution based on the CHHIP protocol and were 1 cm in all directions for PTV1 (PSV), 1 cm/5 mm (posterior) for PTV2 (P) and 5 mm/0 mm (posterior) for PTV3 (P) managing the risk of rectal toxicity [8]. However use of IGRT was not required to compensate for organ motion. We found that random errors produced by intra-fraction (organ motion) and inter-fraction (setup) uncertainties were of similar magnitude. Interestingly, the largest uncertainty was introduced by CTV delineation (LR PC: 1 - 2 mm, IR/HR PC: 1.6 - 3.5 mm). Patient positioning like bone or ERB matching resulted in considerably larger PTV margins [12]. Assuming an optimal delineation error of 1 mm our recommendations for an anisotropic PTV margin are as follows: PTV_P_ 6.2 mm (ant), 5.43 mm (post), 6.06 mm (lng) and 4.79 mm (lat), PTV_PSV_ 8.03 mm (ant), 5.81 mm (post), 7.47 mm (lng) and 6.87 mm (lat). Application of organ tracking would allow for further PTV lowering.

Image-guided patient positioning considerably reduced PTV margin by about 2- 4.7 mm compared with bone or ERB matching which is known from the literature [10]. IGRT modalities such as CBCT and kV/kV-image pairs with FM or CBCT soft tissue matching were interchangeable in respect of accuracy. While some studies have shown the same, others have found that identification of FM on volumetric or planar images was not equivalent (±3 mm) [5]. However, one needs to keep in mind intra-fractional prostate motion between imaging modalities.

In this study, the intra-fractional prostate movement was generally below 2 mm (Σ was 0.36 – 0.75 mm) for treatment duration of around 90 seconds, similar to results from the literature (Σ 1-2 mm) [13]. Prolonged RT duration can increase the intra-fractional prostate movement up to 3-6 mm [14]. Using an ERB might decrease intra-fractional prostate motion. Importantly, the SV move independently from the prostate gland with an uncertainty for PTV between 1.4 – 4.5 mm and should be considered in future margin protocols [15].

Remarkably, the largest uncertainties for PTV margins were produced by the radiation oncologist’s CTV delineation, particularly in the longitudinal axis. In the literature, the inter-observer variation for contouring has been reported to be largest in regions near the seminal vesicles and the apex [16]. Several geometrical uncertainties are involved in the delineation process including imaging resolution [6]. CTV delineation variation can be significantly reduced using MRI, teaching/protocols or automated model-based organ segmentation [17].

In practice, for protocols such as RTOG 0938 trial, a randomized phase II trial on hypofractionated radiation therapy, PTV was defined as the CTV_p_ plus 5 mm in all dimensions except 3 mm posteriorly or anteriorly if necessary [18]. However, reducing treatment margins based on improved treatment accuracy may lead to geographical miss and serious underdosage of the CTV [7]. In order to prevent a geographical miss adaptive IGRT (-2.5-4.5 mm) might be recommended with daily imaging, especially in hypofractionation protocols [19].

The preference of IGRT modality is subject to various considerations. In our study we have chosen FM match in CBCT as reference since the stability of implanted markers was shown by several studies with an average seed migration of 1.2 +/- 0.2 mm (seeds) and 0.8 mm +/- 0.6 mm (coils) [20]. Advantages of kV/kV image pairs compared to CBCT are reduced acquisition/matching time, imaging dose and lower cost. However, it is an invasive procedure with the risk for prostate infection. In our opinion, FM and CBCT complement each other, especially in view of adaptive and real-time tumor tracking [21].

## Consent

Written informed consent was obtained from the patient for data evaluation and publication; in case of participation in the CHIPP trial an additional informed consent was obtained.
